# Associations Between Anxiety, Body Mass Index, and Sex Hormones in Women

**DOI:** 10.3389/fpsyt.2019.00479

**Published:** 2019-07-04

**Authors:** Daniela Stanikova, Tobias Luck, Alexander Pabst, Yoon Ju Bae, Andreas Hinz, Heide Glaesmer, Juraj Stanik, Julia Sacher, Christoph Engel, Cornelia Enzenbach, Kerstin Wirkner, Uta Ceglarek, Joachim Thiery, Juergen Kratzsch, Steffi G. Riedel-Heller

**Affiliations:** ^1^Institute of Social Medicine, Occupational Health and Public Health, University of Leipzig, Leipzig, Germany; ^2^Institute of Experimental Endocrinology, Biomedical Research Center, Slovak Academy of Sciences, Bratislava, Slovakia; ^3^Department of Pediatrics, Medical Faculty, Comenius University and National Institute of Children’s Health, Bratislava, Slovakia; ^4^Department of Economic and Social Sciences & Institute of Social Medicine, Rehabilitation Sciences and Healthcare Research (ISRV), University of Applied Sciences Nordhausen, Nordhausen, Germany; ^5^LIFE-Leipzig Research Center for Civilization Diseases, University of Leipzig, Leipzig, Germany; ^6^Institute of Laboratory Medicine, Clinical Chemistry and Molecular Diagnostics, University of Leipzig, Leipzig, Germany; ^7^Department of Medical Psychology and Medical Sociology, University of Leipzig, Leipzig, Germany; ^8^Center for Pediatric Research Leipzig, University Hospital for Children & Adolescents, University of Leipzig, Leipzig, Germany; ^9^Department of Neurology, Max Planck Institute for Human Cognitive and Brain Sciences, Leipzig, Germany; ^10^Emotion & NeuroimaGinG (EGG) Lab, Max Planck Institute for Human Cognitive and Brain Sciences, Leipzig, Germany; ^11^Clinic of Cognitive Neurology, University of Leipzig, Leipzig, Germany; ^12^Institute for Medical Informatics, Statistics and Epidemiology, University of Leipzig, Leipzig, Germany

**Keywords:** anxiety, body mass index, obesity, sex hormones, testosterone, estrogen, women

## Abstract

**Background:** Several studies have shown a positive association between anxiety and obesity, particularly in women. We aimed to study whether sex hormone alterations related to obesity might play a role in this association.

**Patients and methods:** Data for this study were obtained from a population-based cohort study (the LIFE-Adult-Study). A total of 3,124 adult women (970 premenopausal and 2,154 postmenopausal) were included into the analyses. The anxiety symptomatology was assessed using the GAD-7 questionnaire (cut-off ≥ 10 points). Sex hormones were measured from fasting serum samples.

**Results:** We did not find significant differences in anxiety prevalence in premenopausal obese women compared with normal-weight controls (4.8% vs. 5.5%). Both obesity and anxiety symptomatology were separately associated with the same sex hormone alteration in premenopausal women: higher total testosterone level (0.97 ± 0.50 in obese vs. 0.86 ± 0.49 nmol/L in normal-weight women, *p* = 0.026 and 1.04 ± 0.59 in women with vs. 0.88 ± 0.49 nmol/L in women without anxiety symptomatology, *p* = 0.023). However, women with anxiety symptomatology had non-significantly higher estradiol levels than women without anxiety symptomatology (548.0 ± 507.6 vs. 426.2 ± 474.0 pmol/L), whereas obesity was associated with lower estradiol levels compared with those in normal-weight group (332.7 ± 386.5 vs. 470.8 ± 616.0 pmol/L). Women with anxiety symptomatology had also significantly higher testosterone and estradiol composition (*p* = 0.006). No associations of sex hormone levels and BMI with anxiety symptomatology in postmenopausal women were found.

**Conclusions:** Although both obesity and anxiety symptomatology were separately associated with higher testosterone level, there was an opposite impact of anxiety and obesity on estradiol levels in premenopausal women. We did not find an evidence that the sex hormone alterations related to obesity are playing a significant role in anxiety symptomatology in premenopausal women. This could be the explanation why we did not find an association between obesity and anxiety. In postmenopausal women, other mechanisms seem to work than in the premenopausal group.

## Introduction

Anxiety disorders affect 25% of the population in the Western world, which makes them the most frequent psychiatric condition these days (1). They are chronic and more prevalent in females than males and are typically complicated by coexistent mental and somatic disorders (2). Moreover, there are also differences in comorbidities, symptoms, and how these disorders affect each gender (3).

Differences in prevalence, as well as the fact that puberty, pregnancy, and menopause are important precipitants for onset, exacerbation, recurrence, and relapse of anxiety disorders, further indicate an important role of hormones, including and in particular sex hormones (2).

As for hormones, testosterone is reported to have a crucial influence on the course of anxiety disorders (4). Testosterone, often referred as a male hormone, is also present in women, although in about 10 times lower concentrations (5). It is hypothesized that its higher concentrations in males might be one of the reasons for the sex differences in prevalence of anxiety (4). The effects of testosterone are mediated through the stimulation of estrogen receptors, androgen receptors, or GABAA receptors in several brain regions, resulting in various biological outcomes (6). It has been reported in the literature that testosterone can relieve anxiety and depression while inducing subjective feeling of an improved mood in both genders (2).

Testosterone’s anxiolytic (7, 8) and anxiogenic (9, 10) effects have been studied in several animal studies as well. Various activational effects of testosterone on the anxiety-like behavior have been observed in adult male rodents. Gonadectomized adult male rodents showed increased anxiety-like behavior, and this effect could be reversed by testosterone replacement (11). Moreover, it was shown that testosterone replacement had the exact same anxiety-relieving effect as the administration of the typical tricyclic antidepressant in male rats (12). However, very inconsistent outcomes of studies investigating testosterone’s anxiolytic effect in female rodents have been published. Some studies provide supporting evidence for a beneficial role of testosterone in intact adult female rodents (13).

Women with anxiety disorders were previously proved to have decreased testosterone levels. When compared with healthy controls, women with social phobia, generalized anxiety, or agoraphobia produced lower levels of testosterone in their saliva (14).

On the other side, also an association between higher testosterone levels and anxiety has been shown in women with polycystic ovarian syndrome (state of hyperandrogenism) (15).

Contradictory results from preclinical and clinical research were published regarding the association of anxiety with estrogen levels as well (16, 17). Higher estrogen levels were significantly associated with anxiety and anxiety-like behaviors in some studies (18). The risk for developing of any anxiety disorder significantly increases at menarche, as the circulating estradiol abruptly increases from prepubertal to its adult levels during the stage of sexual development (19).

On the other hand, increased anxiety symptoms were also noticed in women after surgical or natural menopause (20) and close to the end of the luteal phase of the menstrual cycle (21) – periods characterized by decline in circulating estradiol levels. The most probable explanation is that the effect of estradiol on anxiety behavior is dose dependent and varies from anxiogenic (22) to null (23) to anxiolytic (24).

Several conditions might be associated with changes in sex hormone levels in women. The most prevalent is obesity, which might be associated with changes in hypothalamic–hypopituitary–gonadal axis and also altered testosterone and estradiol levels (25). This is also due to the fact that fat tissue *per se* represents an intracrine source of sex hormones, and this was shown to be gender specific. Body mass index (BMI) is positively associated with testosterone levels in women, while there is an inverse association of BMI and estrogen (26). It has been reported that severity of obesity is directly linked with the amplitude of hormonal changes (27), which can be reversed by the reduction of weight (28).

A positive association between overweight and anxiety disorders has been shown in several studies (29, 30), although the underlying mechanisms of this association are still not completely understood. Alterations of testosterone levels (higher levels in women) associated with increased BMI might represent one of the possible risk factors in the etiopathogenesis of anxiety.

We hypothesized that all three factors – anxiety, BMI, and sex hormones – might be strongly interconnected. To the best of our knowledge, all three factors have not been studied together so far.

In the present study, we (1) focused on associations between anxiety symptomatology and BMI, (31) focused on associations between anxiety symptomatology and sex hormones and to compare them with sex hormone alterations in obesity, and (2) studied the impact of both BMI and altered sex hormone levels on anxiety in one regression model.

## Patients and Methods

### Study Population

Data presented in this study were obtained from the (LIFE)-Adult-Study conducted by the Leipzig Research Centre for Civilization Diseases. This study included >10,000 participants selected randomly. The objective of the LIFE-Adult-Study is to investigate the prevalence, markers of early onset, genetic factors, and lifestyle determinants of major civilization diseases, including metabolic diseases and depression (32). The study was approved by the responsible institutional ethics committee of the Medical Faculty of the University of Leipzig (PV 2016-274-04). All participants provided written informed consent. The data privacy and safety concept of the study were endorsed by a responsible data protection officer.

### Medical History and Medications

A structured interview was performed in all participants of the study, where they were asked about 70 common medical diagnoses, which were previously confirmed by their physician. In women, data on menstrual cycle [number of days/months/years since the last menstrual period (LMP)], history of bilateral oophorectomy and hysterectomy, and past or present use of contraception pills or hormonal replacement therapy were included. Data on all other medications taken within past 7 days before study day were gathered. Medications were identified by bar codes, following ATC classification.

### Anthropometry

Trained study personnel measured the body weight and height according to standardized protocols. For measuring body weight, an electronic scale (SECA 701, Seca Gmbh & Co KG) with a precision of 0.01 kg was used; body height was assessed by means of a stadiometer (SECA 240) to the nearest 0.1 cm (32). Underweight was assessed as BMI < 18.5 kg/m^2^, normal weight as BMI ≥ 18.5 and < 25 kg/m^2^, overweight as BMI ≥ 25 kg/m^2^ (pre-obesity BMI > 25 and 29.9 kg/m^2^ and obesity ≥ 30 kg/m^2^).

### Hormonal Analyses

Blood samples were collected in all participants of the study between 7:30 and 10:30 a.m. after more than 10 hours of fasting). Analyses of levels of selected hormonal (sex hormone-binding globulin and total testosterone) and biochemical (albumin) parameters (for calculation of free testosterone) were performed on fresh serum samples in a highly standardized manner (32). Biochemical analysis was performed by fully automated Cobas system (Roche, Mannheim). Intra-assay and inter-assay coefficients of variation were given exemplarily by 100 subsequent analyses during the time of recruitment study of probands: results of sex hormone-binding globulin were <3.4% for the range between 25.4 and 54.2 nmol/L, results of estradiol were below 4.3% for the range between 387 and 2,052 pmol/L, and results of total testosterone were below 4.9% for the range between 3.73 and 19.07 nmol/L. Free testosterone was calculated using Vermeulen’s formula (33). Composition of estradiol and total testosterone or free testosterone was calculated by multiplying serum estradiol levels by total or free testosterone serum levels, respectively.

### Assessing of Anxiety Symptomatology

We used a German version of the established self-report questionnaire Generalized Anxiety Disorder 7 (GAD-7) to screen the presence of anxiety symptomatology. The GAD-7 questionnaire (covering symptoms of generalized anxiety disorder, panic disorder, social anxiety disorder, and post-traumatic stress disorder) consists of seven items asking patients how often, during the last 4 weeks, they were bothered by each symptom. The answer options were “not at all,” “several days,” “more than half the days,” and “nearly every day” scored from 0 to 3 points. A total score of ≥10 indicates the presence of an anxiety symptomatology (34).

### Exclusion Criteria

We excluded all women 1) using medications possibly influencing either hormonal or mental status, including 1A) external hormones [ATC groups: G02, other gynecologicals (*n* = 210); G03, sex hormones and genital modulators (*n* = 766); H02, systemic corticosteroids (*n* = 109); and L02, endocrine therapy (*n* = 60)], 1B) CNS or psychotropic drugs [N03, antiepileptics (*n* = 143); N04, antiparkinsonics (*n* = 61); N05, psycholeptics (*n* = 344); N06, psychoanaleptics (*n* = 516); and N07, other CNS drugs (*n* = 61)]; (31) individuals with hypothyreosis or hyperthyreosis (*n* = 11); 2) perimenopausal (6–12 months since LMP, *n* = 136) and postpartal (1 year after delivery, *n* = 28) women, as both periods are significantly related to increased onset and prevalence of anxiety disorders (35); 3) underweight individuals (*n* = 40) because of small numbers; 4) all individuals with severe renal/hepatal/neurologic disease or cancer in the last year (*n* = 276); and 5) individuals diagnosed or/and treated for depression (*n* = 462).

### Classification Process

Women were classified according to age and LMP into premenopausal (0–6 months since LMP or <45 years) and postmenopausal (>12 months since LMP or ≥55 years or bilateral oophorectomy), in accordance with guidelines used by Breast Cancer Consortium (36).

### Cohort Description

The study sample included 3,124 women of whom 970 (31.0%) were premenopausal (45.0 ± 6.6 years) and 2,154 (68.9%) were postmenopausal (64.2 ± 8.0 years). In the group of premenopausal women, 53.7% were of normal-weight (mean BMI 22.1 ± 1.6 kg/m^2^), 28.1% were pre-obese (mean BMI 27.1 ± 1.4 kg/m^2^), and 17.0% were obese (mean BMI 35.2 ± 5.0 kg/m^2^). In the group of postmenopausal women, 32.5% were normal weight (mean BMI 22.8 ± 1.6 kg/m^2^), 37.9% were pre-obese (mean BMI 27.4 ± 1.4 kg/m^2^), and 29.0% were obese (mean BMI 34.2 ± 4.1 kg/m^2^).

### Statistical Analyses

Values for the sample description are given as mean ± SD. Comparison between groups was tested using *t*-test for metric variables and by Fisher’s test or chi-square test for binary variables. Logistic regression analysis was performed using presence of anxiety symptomatology (GAD-7 ≥ 10 points) as a dependent variable, and BMI, testosterone, and age as independent variables. *p* values less than 0.05 were considered as statistically significant. Statistical analyses were performed with SPSSv25 software (IBM, NY, USA). Graphs were plotted with GraphPad Prism 7.04 (GraphPad Software, CA, USA).

## Results

### Prevalence of Anxiety in Women

**Figure 1:**
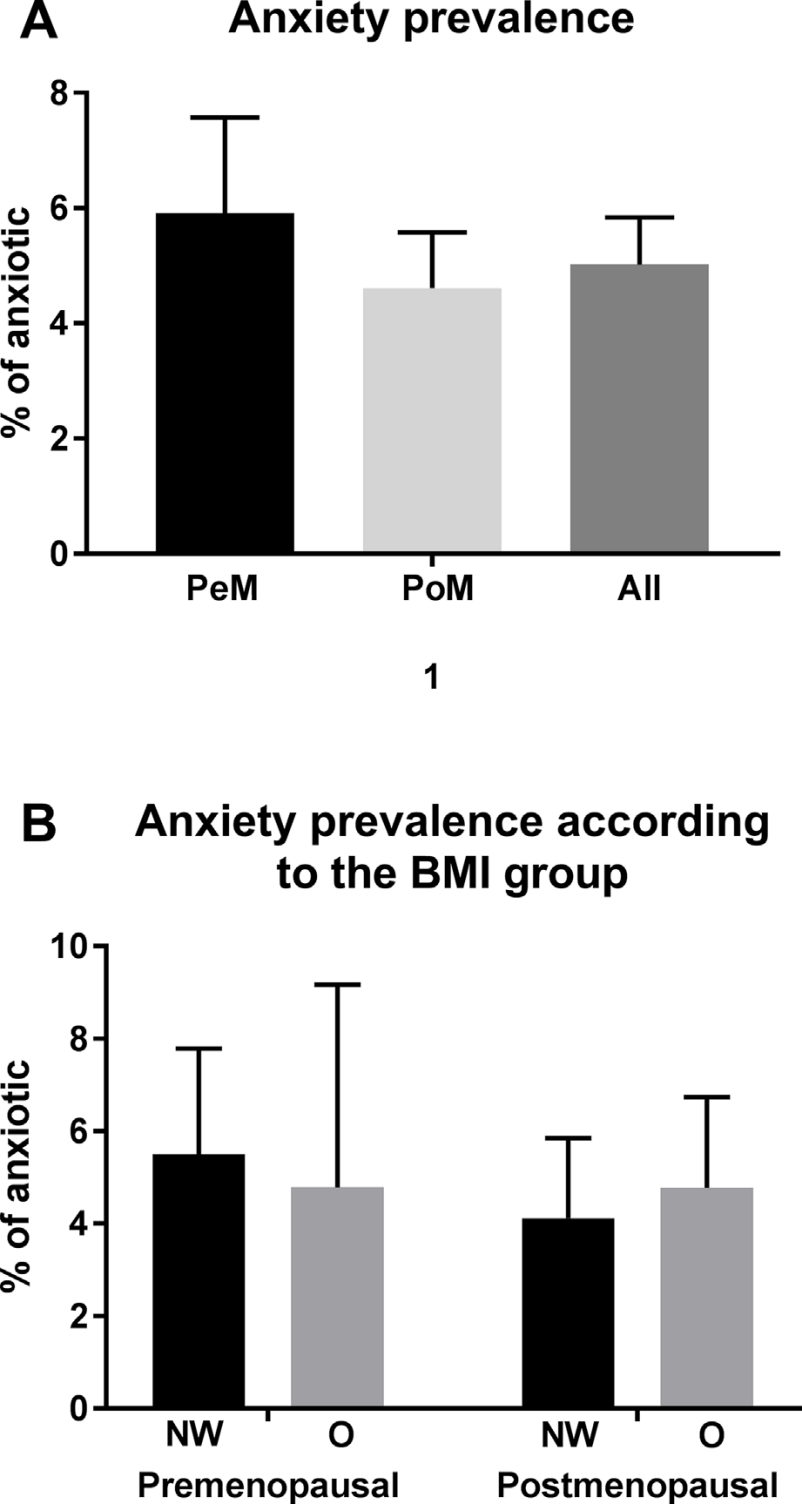
Prevalence of anxiety. Displayed in % with 95% confidential intervals (calculated with the Wilson–Brown method). Significant differences are marked with * (*p* between 0.05 and 0.01), ** (*p* between 0.01 and 0.001), and *** (*p* < 0.001). Abbreviations: PeM, premenopausal; PoM, postmenopausal.

In all women included into the study, the prevalence of anxiety symptomatology was 5.0%, with no significant differences between the premenopausal and postmenopausal groups (**Figure 1A**).

### Association Between Anxiety Symptomatology and BMI

We did not find any significant differences in the prevalence of anxiety symptomatology between normal weight, and obese premenopausal, postmenopausal women (**Figure 1B**).

### Associations of Anxiety Symptomatology and BMI with Sex Hormones

**Figure 2:**
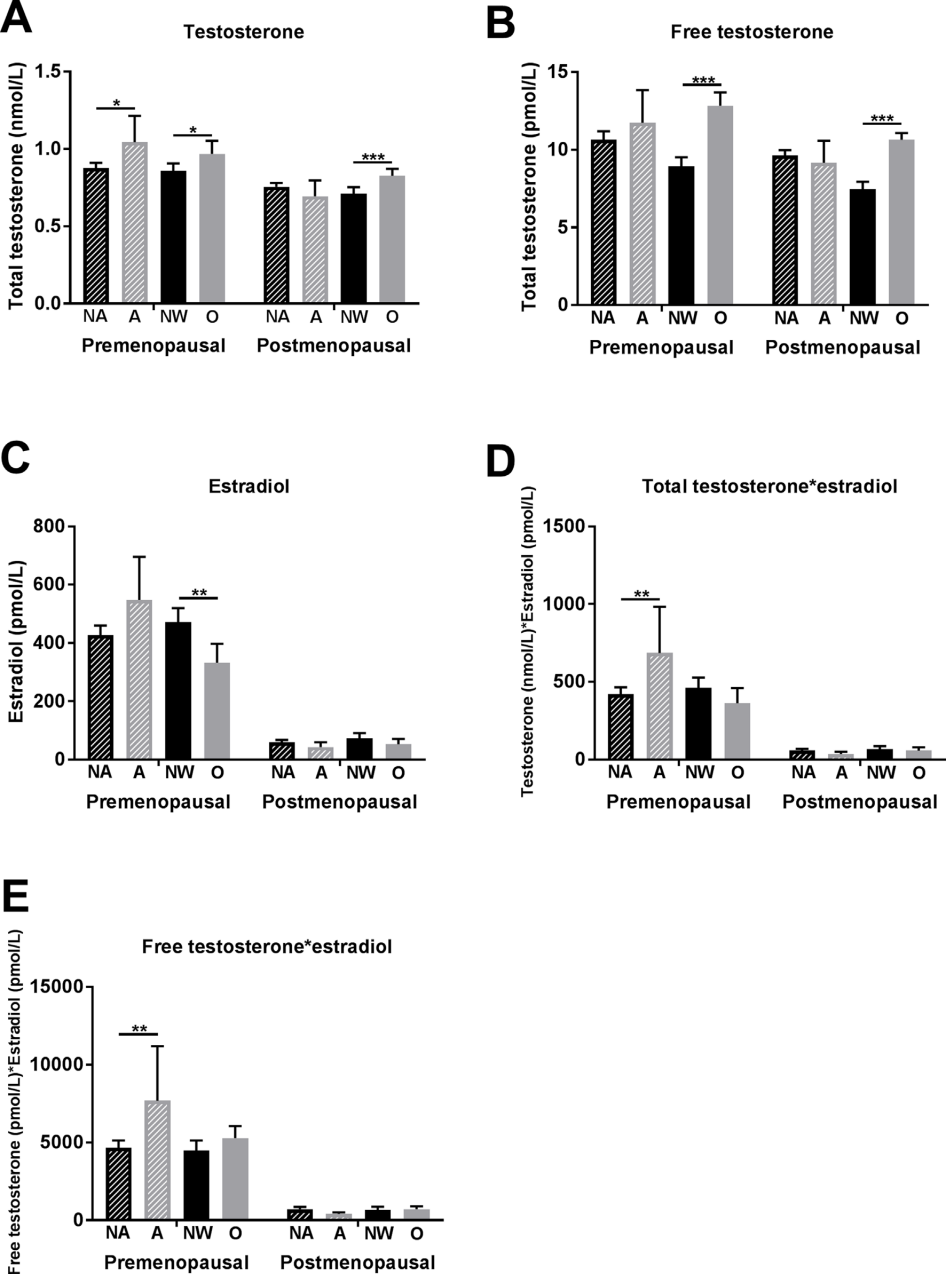
Associations of anxiety symptomatology and obesity with testosterone and estradiol levels in premenopausal and postmenopausal women. **(A)** Total testosterone levels, **(B)** free testosterone levels, **(C)** estradiol levels, **(D)** composition of total testosterone with estradiol levels, and **(E)** composition of free testosterone with estradiol levels. Significant differences are marked with * (*p* between 0.05 and 0.01), ** (*p* between 0.01 and 0.001), and *** (*p* < 0.001). Abbreviations: NA, non-anxious (anxiety symptomatology not present = GAD-7 < 10 points); A, anxious (anxiety symptomatology present = GAD-7 ≥ 10 points); NW, normal weight (BMI between 18.5 and 25 kg/m^2^); O, obese (BMI ≥ 30 kg/m^2^).

Premenopausal women with anxiety symptomatology showed higher total testosterone levels than did women without anxiety symptomatology (1.04 ± 0.59 vs. 0.88 ± 0.49 nmol/L, *p* = 0.023) (**Figure 2A**). Free testosterone and estradiol levels were also higher in the women with presence of anxiety symptomatology; however, the differences were not significant (**Figure 2B, C**). Significant differences in anxious versus non-anxious women were observed in compositions of estradiol with total and free testosterone, respectively (**Figure 2D, E**).

In postmenopausal women, no significant association between anxiety symptomatology and testosterone (as well as with other sex hormones) was found (**Figure 2A–E**).

Premenopausal women with obesity showed higher total testosterone (0.97 ± 0.50 vs. 0.86 ± 0.49 pmol/L, *p* = 0.026) and free testosterone levels (12.8 ± 8.3 vs. 8.9 ± 6.2 pmol/L, *p* < 0.001) and lower estradiol levels (332.7 ± 386.5 vs. 470.8 ± 516.0 pmol/L, *p* = 0.004) than did normal-weight women (**Figure 2A–C**).

### Association Between Anxiety Symptomatology, BMI, and Sex Hormones

In the logistic regression analysis, anxiety symptomatology was significantly associated with total testosterone (∆*R* ^2^ = 0.016, OR = 1.773, *p* = 0.025), but not BMI (**Table 1**).

**Table 1:** Logistic regression analysis of anxiety symptomatology and co-factors in women. Anxiety symptomatology (assessed as 0–1, cut-off GAD-7 ≥ 10 points) was set as dependent variable. Independent variables were total testosterone, estradiol, and BMI. Stepwise model was used for all analyses. Abbreviations: BMI, body mass index; ∆*R* ^2^, *R* square change; OR, odds ratio; CI, confidence intervals; *n*, number of analyzed individuals; *p* value, significant if <0.05.

Dependent variable: anxiety symptomatology Independent variables: *total testosterone, estradiol, BMI*	Included independent variable	∆*R*^2^	OR	CI (95%)	*p*
**Premenopausal women**. Model summary: ***R*** **^2^** ** = 0.016,** ***p*** ** = 0.033,** ***n*** ** = 799**	**1. Total testosterone**	0.016	1.763	1.074–2.895	**0.025**
	Not included: estradiol (*p* = 0.230), BMI (*p* = 0.124)				
**Postmenopausal women**. Model summary: ***n*** ** = 1,708**	Not included: total testosterone (*p* = 0.278), estradiol (*p* = 0.423), BMI (*p* = 0.628)				

In postmenopausal women, total testosterone, estradiol, and BMI were not associated with anxiety symptomatology (**Table 1**).

## Discussion

Both anxiety symptomatology and obesity were associated with higher testosterone levels but had opposite impact on the estradiol levels in premenopausal women. However, BMI was not associated with anxiety symptomatology. No significant associations between anxiety symptomatology and sex hormone levels were found in postmenopausal women.

### Anxiety Symptomatology and Testosterone

The positive association between anxiety and testosterone levels in women, found in our study, has been observed in several others, particularly in studies of women with polycystic ovarian syndrome (state of hyperandrogenism) (37, 38). A similar association has also been found in animal studies. In a study by Goel and Bale (9), an anxiogenic effect after administration of testosterone in female mice was observed. Increased androgen levels in prenatal period were proved to be associated with anxiety-like behavior in rats. This effect was reversible by the administration of estrogen receptor modulators and androgen receptor blockers. This study also reported anxiety-like behavior in female rats after they were injected testosterone into the amygdala (39).

Females were proved to have more sensitive receptor binding testosterone despite about 10 times lower levels of testosterone than have males (2, 5). This can be seen also in our study—premenopausal women with anxiety symptomatology had higher total testosterone levels, but the mean levels were still within the reference range. This is implying that women might be sensitive not only to extreme but also to more subtle alterations of testosterone levels. The fact that this association was observed in premenopausal women only might be related to physiological changes of testosterone levels over a woman’s lifespan. Between 20 and 40 years, a steep decline of testosterone levels can be seen in women (40). Premenopausal women could be therefore more sensitive to the (pathophysiological) increase of testosterone level.

On the other hand, it is necessary to say that several studies have found an association between lower testosterone and anxiety (or some specific subtypes) in women (14, 41).

The most possible explanation is that the association between anxiety and testosterone levels might be not linear and that the effect is dose dependent. This was observed in one of the most cited animal study focused on the effect of testosterone on anxiety (42). Differences in the study design (e.g., with regard to the menopausal status of women) as well as studied samples might also play a role in the various outcomes.

### Anxiety Symptomatology and BMI

Several large epidemiologic studies have found a positive relationship between anxiety disorders (or some specific subtypes) and overweight or obesity in women and men, i.e., increasing prevalence with increasing BMI (29). A moderate but positive relationship between overweight and anxiety disorders was also observed in large epidemiological studies and meta-analyses (30, 43).

In our study, no significant association between BMI and prevalence of anxiety symptomatology regardless of gender or menopausal status in women was found.

There might be several possible explanations for these contradictory results. The first possibility is the way that anxiety was measured. Most of the published studies focused on the lifetime or on the 12-month prevalence of anxiety (29, 44), and only a few studies focused on current anxiety symptomatology with current BMI. In the study by Jorm et al. (45) investigating the association between current anxiety symptomatology and current BMI status, similar results to our study have been found. Anxiety symptomatology and BMI are largely time-dependent phenotypes; e.g., higher anxiety symptoms earlier in life may contribute to increased BMI later in life and vice versa. To the best of our knowledge, a study investigating the association between BMI in the age of the onset of anxiety disorder is missing.

Another possible explanation for these contradictory findings of above-mentioned studies and our study is that most of them did not use any exclusion criteria, which may have an impact on the results of anxiety and weight of an individual as well. In our study, by contrast, all individuals treated with psychotropic or selected CNS drugs, as well as with severe somatic conditions (e.g., cancer), were excluded. Also, comorbidity of depression and anxiety is high (29); it could be that the positive association described also in other studies of increased BMI and anxiety could be driven by comorbid depression.

### Anxiety Symptomatology, BMI, and Testosterone

A well-established fact is that increased BMI is associated with higher testosterone levels in women and decreased levels in men (28, 46). First, we hypothesized that there might be a mediation of the positive association between anxiety and overweight (described in several studies) through altered testosterone levels associated with increased BMI. If this hypothesis would be true, this could change the therapy approach of anxiety disorders in obese women, as higher testosterone level might maintain anxiety symptomatology. Therefore, obese women with anxiety symptomatology could benefit from androgen receptor blockers or weight loss as an additive therapy.

However, we did not find a significant association between anxiety symptomatology and BMI in either premenopausal or postmenopausal women in our study. There could be several explanations of this finding:

- Hormonal changes associated with gradually increasing BMI are slow, so a certain “adaptation mechanism” might develop and could explain why the BMI-associated sex hormone alterations were not proven to play a significant role in anxiety pathogenesis in overweight individuals.
- Hormonal changes associated with obesity might be “protective” against anxiety. Therefore, we have looked at the concentrations of other sex hormones. Estradiol levels were significantly lower in obese premenopausal women compared with the normal-weight group, and non-significantly (*p* = 0.086) higher in the group of women expressing anxiety symptomatology than in the women without anxiety symptomatology. Therefore, lower estradiol levels associated with obesity could be protective against anxiety in premenopausal women. To support our hypothesis, we calculated total and free testosterone * estradiol index, as higher levels of both hormones were associated with anxiety. Higher values of this index (using both total and free testosterone) were significantly associated with anxiety symptomatology in premenopausal women, but there were no significant differences between obese and normal-weight groups.
- The association between increased testosterone levels and anxiety symptomatology independent of BMI might indicate that other conditions related to higher testosterone level in women [e.g., stress (47), smoking (48), polycystic ovarian syndrome (37), insulin resistance (49), and hypothyreosis] could be involved in the pathomechanism of anxiety in premenopausal women, and most of these conditions are highly influenceable by lifestyle changes.
- Another speculation could be that anxiety *per se* might lead to increased testosterone levels in women, so this might be secondary to anxiety. Further studies are required to establish causal relationships and to elucidate possible neural and molecular mechanism underlying testosterone’s actions in anxiety.

In postmenopausal women, no association between testosterone and anxiety, as well as anxiety and BMI, was shown in our study. Several other factors (e.g., partnership or being married and socioeconomic status) might play a more significant role in the mechanism of anxiety besides those two factors.

### Strengths and Limitations of the Study

To our best knowledge, this is the first study investigating associations between anxiety, BMI, and sex hormones in women together. It is based on a large cohort of more than 3,000 individuals, where strict exclusion criteria were employed. A further strength is that anthropometric measurements were taken by study personnel rather than self-report, which is related to higher reliability. As a limitation, the cross-sectional nature of the study with a relatively healthy population sample might have hampered the study of these associations. The studied associations might be more pronounced in a sample of patients with clinical diagnosis of anxiety disorder. Further limitation could be the fact that the sex hormone levels were not measured in the same phase of the menstruation cycle. However, our aim was not to study association of the anxiety symptomatology with the menstruation cycle but to study the presence of anxiety symptomatology with levels of sex hormones, which was performed on the same day. Another limitation could be the polycystic ovary syndrome. However, in our cohort, we did not have any female participants labeled with polycystic ovary syndrome that was associated with anxiety in several studies (37).

## Conclusions

Although both obesity and anxiety symptomatology in premenopausal women were separately associated with the same hormonal phenotype – higher testosterone levels – there was an opposite impact of anxiety and obesity on estradiol levels. This could be the explanation why we did not find a relationship between anxiety symptomatology and obesity. This suggests that there is no direct link between anxiety, BM, and sex hormones and that sex hormone alterations in obese women do not play a significant role in developing or maintaining anxiety. As the prevalence of obesity and mental disorders is increasing, further studies investigating other factors associated with anxiety might bring light into the pathomechanism of anxiety in young women. In postmenopausal women, other mechanisms seem to work than in the premenopausal group.

## Data Availability Statement

All datasets generated for this study are included in the manuscript and the supplementary files.

## Ethics Statement

Data included in the study were obtained from the Leipzig Research Centre for Civilization Diseases (LIFE)-Adult-Study. The study was approved by the responsible institutional ethics board of the Medical Faculty of the University of Leipzig (PV 2016-274-04). Written informed consent was obtained from all participants. The data privacy and safety concept of the study were endorsed by the responsible data protection officer.

## Author Contributions

All authors reviewed the manuscript critically and approved the final version. DS was responsible for the conception and design of the study, analysis of the data, and writing of the manuscript. YJB, JK, JT, UC, CEng, and KW acquired the data. TL, JSt, AP, AH, HG, JSa, CEnz, and SR-H contributed to the interpretation of the data and critical revision of the manuscript.

## Funding

This work was supported by LIFE, Leipzig Research Center for Civilization Diseases, at the University of Leipzig. LIFE is funded by means of the European Union, by means of the European Social Fund (ESF), by the European Regional Development Fund (ERDF), and by means of the Free State of Saxony within the framework of the Excellence Initiative. The Integrated Research and Treatment Center Adiposity Diseases is funded by the German Federal Ministry of Education and Research (Grant 01EO1501). JSt at the time of the writing of the manuscript was supported by ESPE (European Society for Pediatric Endocrinology) Research Fellowship. We acknowledge support from the German Research Foundation (DFG) and University Leipzig within the program of Open Access Publishing.

## Conflict of Interest Statement

The authors declare that the research was conducted in the absence of any commercial or financial relationships that could be construed as a potential conflict of interest.

## Abbreviations

GABAA, Gamma-Aminobutyric Acid A; BMI, body mass index.
